# Bibliometric analysis and visualization of adenomyosis: From 1980–2024

**DOI:** 10.1097/MD.0000000000051082

**Published:** 2026-10-16

**Authors:** Qicai Hu, Liping Zeng, Chunlei Guo, Chunying Li, Ruifang Wu, Xiuli Sun

**Affiliations:** a Department of Obstetrics and Gynecology, Peking University People’s Hospital, Beijing, China; b Department of Obstetrics and Gynecology, Peking University Shenzhen Hospital, Shenzhen, China; c Institute of Obstetrics and Gynecology, Shenzhen PKU-HKUST Medical Center, Shenzhen, China; d Health Science Library, Peking University, Beijing, China.

**Keywords:** adenomyosis, bibliometrics, endometrial neoplasms, epithelial-mesenchymal transition, high-intensity focused ultrasound ablation, pregnancy outcome

## Abstract

**Objectives:**

This bibliometric analysis systematically evaluated the research landscape, emerging trends, and key focus areas in adenomyosis to guide future research directions.

**Design:**

Performance analysis combined with scientific mapping of global adenomyosis research literature.

**Data sources:**

Web of Science Core Collection (1980–2024).

**Data extraction and synthesis:**

Articles were manually screened. Bibliometrix (R 4.3.0), VOSviewer, and CiteSpace analyzed productivity, collaboration, citation patterns, and research trends. Derived data and analysis scripts are deposited in the Open Science Framework (OSF) under a citable DOI.

**Results:**

Among 3100 included publications, China led output (752, 24.26%, full-counted), followed by the United States (414, 13.35%), Japan (350, 11.29%), Italy (215, 6.94%), and South Korea (128, 4.13%). In the 2 adenomyosis-specific corpora, however, the United States ranked first. The US had the highest citations and international collaboration, though international co-authorship among top-producing countries remained low (16.14%). Leading institutions were Fudan University (89), Shandong University (52), and University of Tokyo (49). Top journals by volume: Fertility and Sterility, Human Reproduction, and Journal of Minimally Invasive Gynecology. Research focus shifted from hysterectomy and uterine artery embolization toward high-intensity focused ultrasound (HIFU), and from endothelial nitric oxide synthase/induced adenomyosis toward ectopic endometrium. Eight keyword clusters emerged; the 4 largest (#0 EMT, #1 HIFU, #2 endometrial cancer, #3 pregnancy outcomes) form the present focus. Burst analysis identified “consensus” (2020–2024), “pathogenesis” (2022–2024), and “pregnancy outcome” (2022–2024) as recent and currently active hotspots.

**Conclusions:**

Current adenomyosis research centers on ectopic endometrium and EMT in pathogenesis, fertility outcomes, long-term efficacy of HIFU and fertility-sparing surgery, links to endometrial cancer, and adverse pregnancy outcomes. Beyond deepening pathogenesis and pregnancy-outcome research, key future directions include enhancing international collaboration and developing internationally agreed, evidence-based standardized terminology, diagnostic criteria, and classification systems for adenomyosis.

**Key Points**

**Strengths**

- We conducted a systematic bibliometric analysis of international adenomyosis research spanning over 4 decades.
- This study comprehensively examined the publication landscape (including output volume, country and author affiliations, journal distribution) and research themes (covering geographical contributions and thematic focuses) in adenomyosis research.
- Three established bibliometric software tools (Bibliometrix, VOSviewer, CiteSpace) were employed to ensure robust and reproducible analysis.
- A sensitivity analysis across 3 corpora (broad, title-level restrictive, adenomyosis-specific metadata) was performed to assess the robustness of the principal findings to retrieval strategy.
- Derived bibliometric indicators and the full R analysis script are deposited in the Open Science Framework under a citable DOI, supporting independent verification.

**Limitations**

- Relying solely on citation databases (Web of Science Core Collection) for trend analysis in adenomyosis research may underestimate the actual research output, as non-indexed journals and non-English publications were excluded.
- Potential selection biases exist due to the choice of database and the use of different analytical software tools.
- The cross-sectional nature of bibliometric data limits causal inference regarding research impact.
- The 82% hysterectomy figure cited in the Introduction refers specifically to a US insured-population cohort (Kaiser Permanente Washington, 2006–2015) and may not generalize to other healthcare systems or uninsured populations.
- Raw Web of Science Core Collection (WoSCC) exports cannot be redistributed under the WoSCC licence; only derived indicators and the analysis script are openly deposited.

## 1. Introduction

Adenomyosis involves endometrial tissue invading the uterine muscle, causing the uterus to enlarge.^[1,2]^ It features ectopic endometrial glands and stroma within thickened myometrium.^[3]^ Common symptoms include heavy menstrual bleeding, dysmenorrhea, and infertility,^[4]^ severely affecting women’s quality of life and fertility. It also raises the risk of pregnancy complications and negative neonatal outcomes.^[5]^ In a US insured-population cohort (Kaiser Permanente Washington, 2006–2015) that excluded women with prior hysterectomy, 82% of women with adenomyosis eventually underwent hysterectomy.^[6]^ Hysterectomy thus remains a major component of the disease burden in this population, although generalizability to other healthcare systems and uninsured populations is limited.

In recent decades, research on adenomyosis has witnessed rapid development, accompanied by a substantial increase in the volume of related literature. The research scope has broadened substantially, encompassing several key areas: from studies comparing the diagnostic efficacy of MRI versus transvaginal ultrasound,^[7]^ to analyses of correlations between imaging features and histopathological characteristics^[8]^; from investigations into the highly heterogeneous nature of its clinical presentation,^[9,10]^ to in-depth research on underlying pathogenic mechanisms^[11,12]^; and from descriptions of classification criteria and features for different subtypes,^[13,14]^ to challenging analyses concerning diagnostic approaches^[15,16]^ and clinical management strategies.^[17]^ However, systematic statistical collation and visual mapping remain lacking.

Bibliometrics is a method involving the statistical analysis of scientific publications, using techniques such as co-citation, citation, and keyword analysis to uncover research trends and inform future studies.^[18]^ This study applies bibliometric methods to the adenomyosis research field, examining annual publication volume, geographical output distribution, single-country versus international collaborations, institutional and author contributions, journal patterns, and reference and keyword analyses. The goal is to comprehensively map the progress in adenomyosis research and provide a scientific basis for a deeper understanding of its evolutionary trends, current landscape, and potential hotspots.

## 2. Materials and methods

### 2.1. Data sources and search strategy

The data for this study were sourced from the Web of Science Core Collection (WoSCC). Recognized as one of the most comprehensive platforms for scientometric analysis, WoSCC enables the identification of emerging research trends and supports in-depth analyses based on citation data. The search strategy was designed as follows: TS=(“adenomyo*”) OR TS=(“endometriosis of the uterus”) OR TS=(“uter* endometriosis”) OR TS=(“internal endometriosis”). The search encompassed publications from January 1, 1980, to December 31, 2024, and was conducted on 30 January 30, 2025. The year 1980 was selected as the starting point because it corresponds to the earliest records of adenomyosis-related publications indexed in WoSCC and marks the beginning of the modern era of systematic adenomyosis research following foundational pathological descriptions. Subsequently, the retrieved records were preliminarily filtered using the WoSCC online filter tool. The following types of publications were excluded: documents that were not articles or reviews; publications in languages other than English; and publications categorized under Web of Science categories that fall outside clinical and basic biomedical research – specifically, Agriculture Dairy Animal Science, Plant Sciences, Veterinary Sciences, and Marine & Freshwater Biology (Fig. 1). To ensure data quality, 3 independent reviewers (Qicai Hu, Chunlei Guo, and Liping Zeng) conducted further screening of the retrieved records by examining titles, abstracts, and full texts to exclude publications deemed irrelevant to the research topic. Inter-rater agreement for the 3 raters was assessed with Fleiss’ κ, yielding κ = 0.71 (95% CI: 0.66–0.76); discrepancies were resolved through discussion and consensus. (Pairwise Cohen κ values are reported in the screening log archived alongside the deposited analysis script.) Finally, the screened literature was exported in plain text (txt) files, containing both full records and cited references, for subsequent bibliometric and visualization analyses.

**Figure 1.**
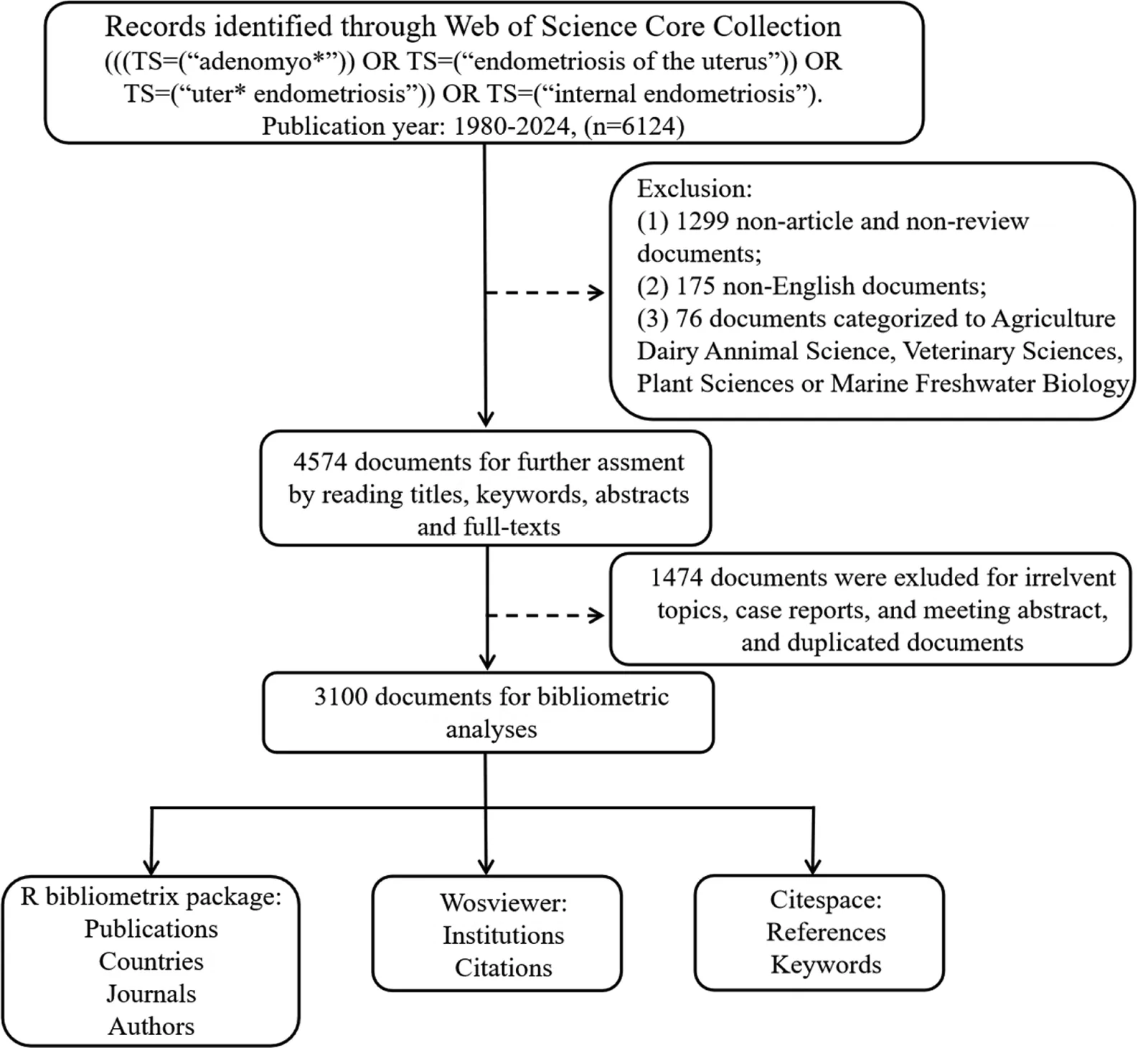
Flowchart of article inclusion for bibliometric analysis.

### 2.2. Data analysis

Bibliometric indicators and network analyses were performed using the Bibliometrix R package (version 4.3.0; R version 4.3.0),^[18]^ with VOSviewer (version 1.6.20) and CiteSpace (version 6.4.R1) providing complementary visualization. The main statistical indicators included the number of articles published, country/region distribution, journal distribution, author contribution, local citation score (LCS), impact factor, H-index, g-index, number of references, annual growth rate, and average number of citations per article. Author productivity metrics are based on raw WoS author name strings. Given that WoS does not assign unique author identifiers, homonymous authors may be merged, and the same author may appear under different name variants (e.g., “Sunwei Guo” and “Guo SW”). Therefore, all author-level findings should be interpreted as “author-name string productivity” rather than individual investigator-level performance. Country contribution was analyzed using full counting. Full-counted country percentages do not sum to 100% across the dataset, as multi-country publications are counted for each contributing country. This method differs from fractional counting or corresponding-author-based analyses. Collaborative networks between countries were also analyzed. Prior to keyword co-occurrence analysis, a harmonization step was performed. The following generic anatomical, demographic, and methodological terms were excluded from the thematic dictionary as background noise: “women,” “patient,” “uterus,” “uterine,” “human,” “female.” Terms referring to adjacent disease categories (e.g., “leiomyoma,” “endometriosis”) were retained and flagged as adjacent-topic terms in Table S4, Supplemental Digital Content 1. Journal impact factors were sourced from the 2023 Journal Citation Reports (JCR).

VOSviewer (version 1.6.20), widely employed in multidisciplinary bibliometric analyses due to its capacity for efficiently processing large-scale datasets, was utilized in this study to visualize the country collaboration network (Fig. 3C). Total Link Strength (TLS), as reported in Table 1, is defined as the sum of link weights between a node and all its connections in the VOSviewer-rendered network; TLS values in Table 1 were calculated by VOSviewer v1.6.20 and exported to the table. VOSviewer network parameters for the country collaboration map (Fig. 3C) were as follows: the network included only the top 50 most productive countries (Top N = 50) and applied a minimum-link threshold of 5 collaborative publications per country edge; full counting was used for all link weights. Note: VOSviewer does not support the concept of per-slice rendering as in CiteSpace; accordingly, the country collaboration map was generated as a single network across the full study period, not in temporal slices.

**Table 1:** Top 10 productive institutions ranked by the number of publications related to adenomyosis.

Rank	Institutions	Counts	Country	H-index	TLS	Total citation	Average citation per year
1	Fudan University	89	China	27	3101	2225	49.44
2	Shandong University	52	China	22	1066	631	14.02
3	The University of Tokyo	49	Japan	26	743	818	18.18
4	Capital Medical University	47	China	18	920	707	15.71
5	Chongqing Medical University	43	China	20	580	963	21.40
6	Shanghai Jiao Tong University	42	China	18	1214	884	19.64
7	Zhejiang University	42	China	17	663	486	10.80
8	University of Siena	41	Italy	28	2671	2588	57.51
9	Catholic University Louvain	38	Belgium	22	1274	2105	46.78
10	University Roma Tor Vergata	37	Italy	23	1912	1991	44.24

H-index values in this table represent field-specific H-index calculated for adenomyosis research only, and may differ from the institutions’ overall H-index across all disciplines.

TLS = total link strength.

CiteSpace (version 6.4.R1), a Java-based bibliometric analysis tool, supports citation analysis, co-authorship analysis, and keyword co-occurrence analysis. Through analyses of keyword clustering, burst detection, betweenness centrality, and heterogeneous networks, CiteSpace effectively identifies research hotspots and emerging trends within a field.^[19]^ In this study, CiteSpace was used to perform co-citation clustering and keyword analysis (Figs. 4 and 5). For the sensitivity analysis reported in Table S3, Supplemental Digital Content 2, the 3 corpora were constructed concurrently, and their bibliometric indicators retrieved using a single Web of Science Core Collection snapshot to ensure internal consistency across comparison groups.

**Figure 2.**
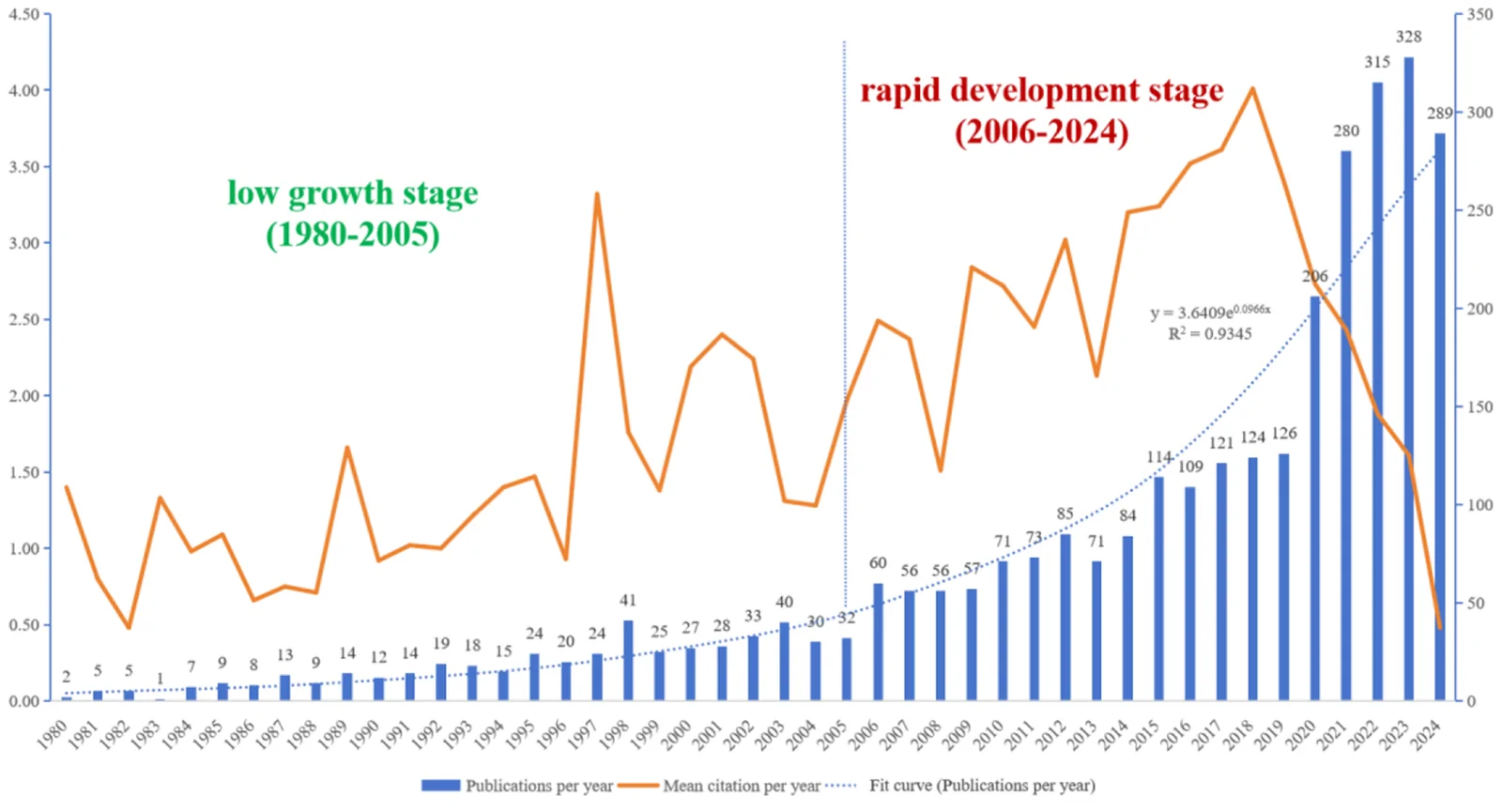
The annual number of publications and mean citation per year on adenomyosis from 1980 to 2024 (Data are based on the full 3100-record broad corpus, using full counting).

**Figure 3.**
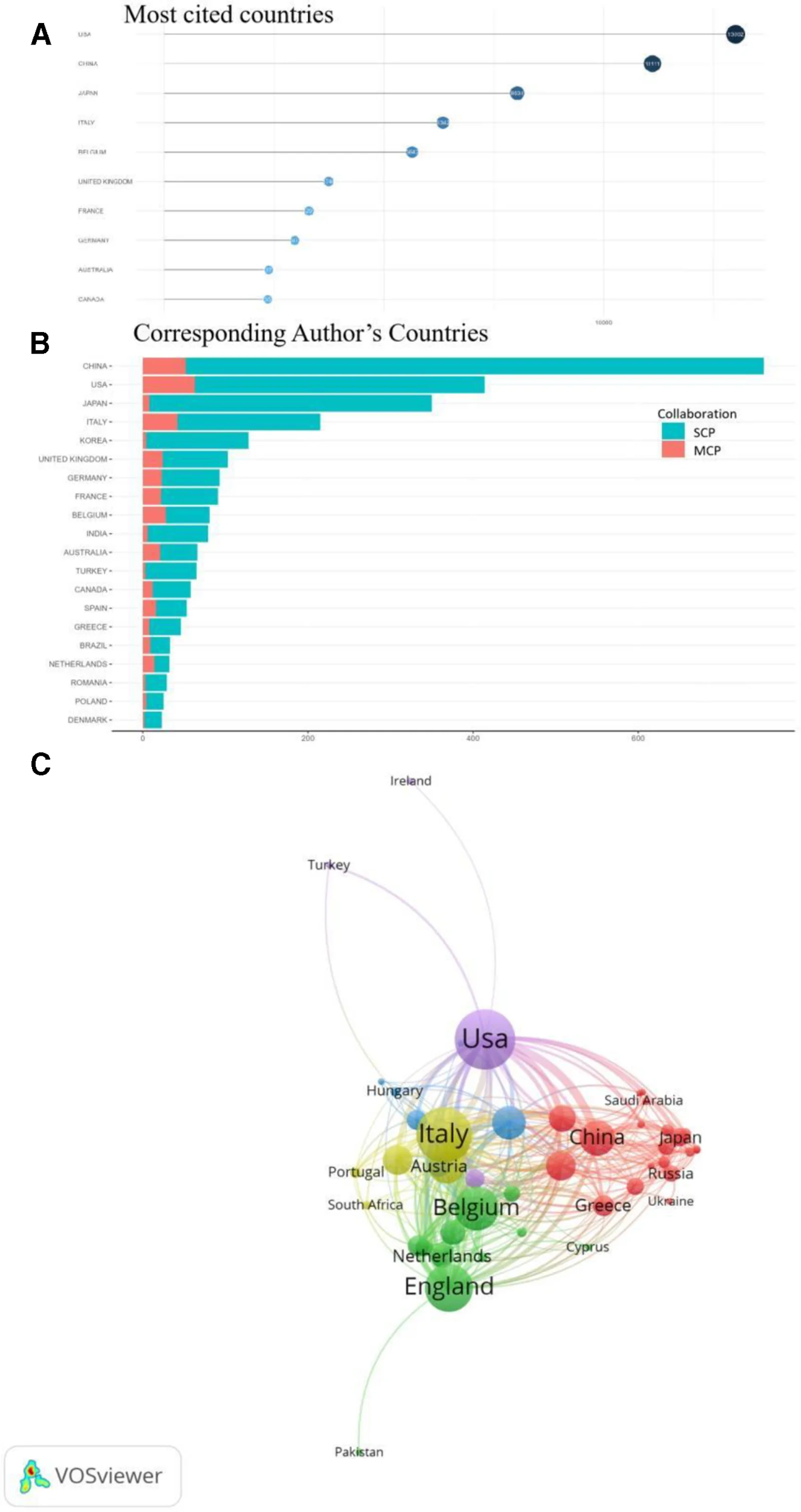
Analyses of country in adenomyosis. (A) The top 10 most cited countries (Data use full-counted author-address countries). (B) Statistics of collaborative publications (Data use full-counted author-address countries; SCP = single-country publication, MCP = multi-country publication). (C) The countries’ collaboration network visualization map (Network is based on full-counted collaboration links between countries with ≥5 collaborative publications).

**Figure 4.**
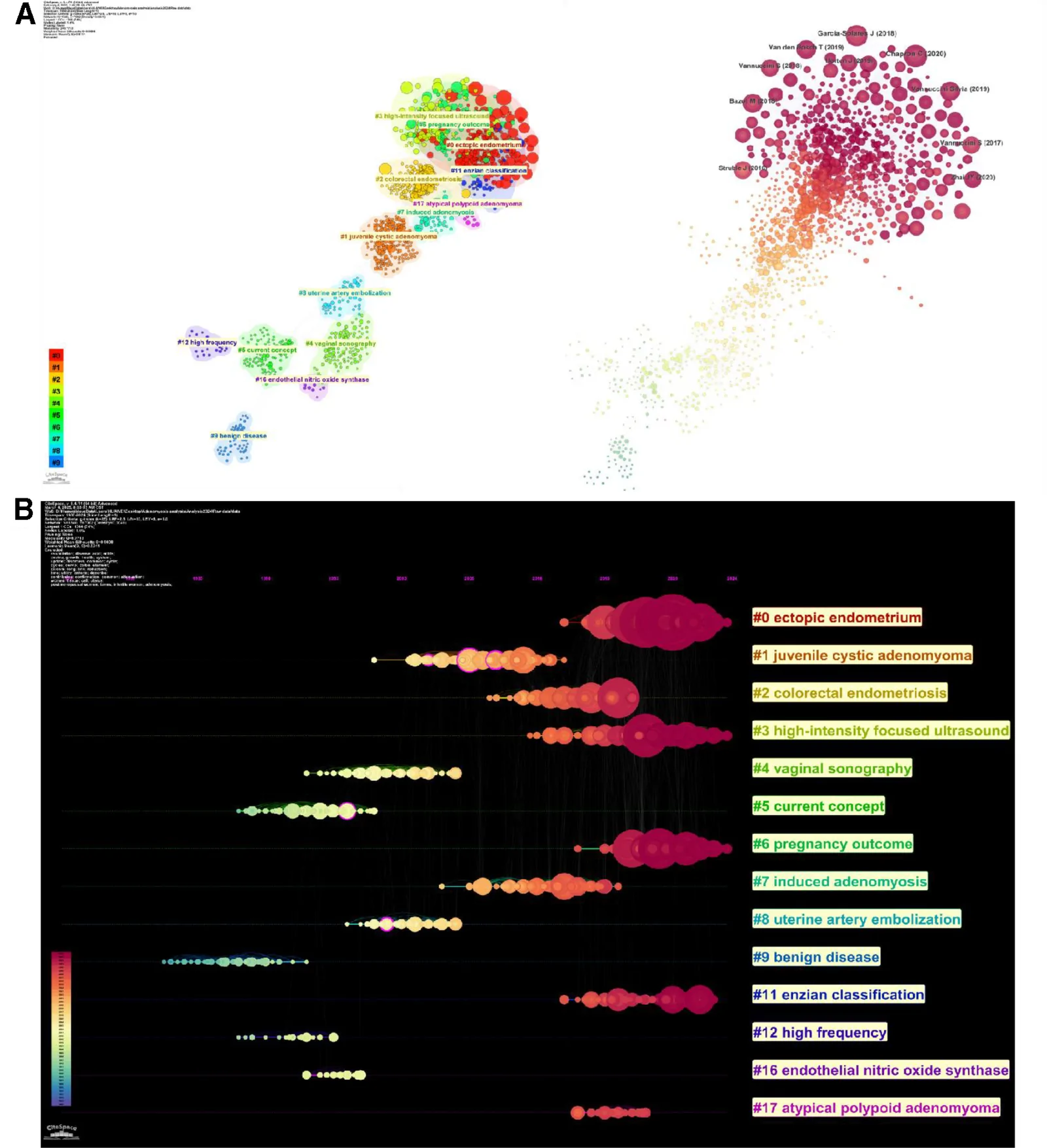
Visualization map of co-cited references. (A) Cluster view; (B) timeline view. The time evolution is indicated by different colored lines, and the nodes on the lines indicate the cited references. Data layer: the network is based on co-cited references in the full 3100-record corpus; in panel B, node position reflects the year of citation.

**Figure 5.**
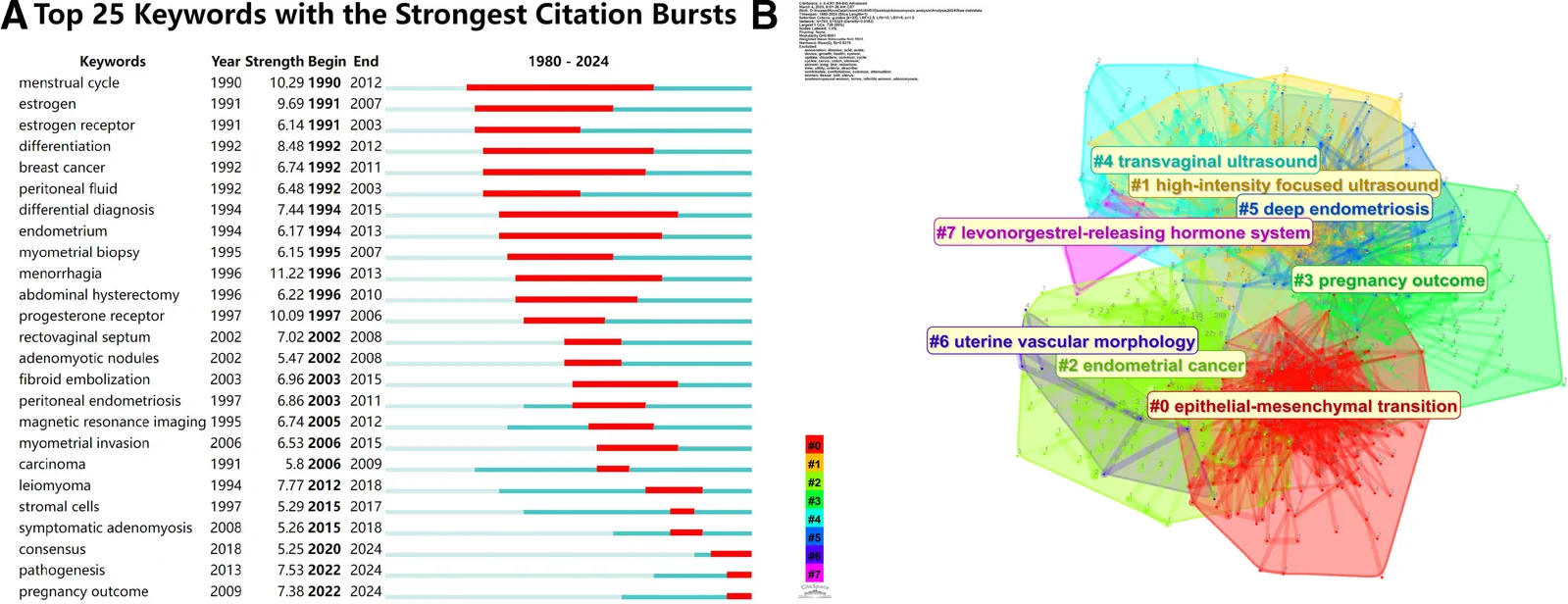
Keyword visualization. (A) The top 25 keywords with the strongest citation bursts from 1980 to 2024 (Burst detection is based on harmonized author keywords and Keywords Plus from the full 3100-record corpus). (B) Cluster mapping of keywords; different colors represent different clusters (Clustering is based on harmonized author keywords and Keywords Plus, after exclusion of generic background terms; 8 clusters are identified, of which the 4 largest [#0–#3] are discussed in the text).

### 2.3. CiteSpace parameter settings

CiteSpace parameters: Time slicing was set to 1 year per slice to capture the fine-grained temporal evolution of research topics. Node selection was based on the g-index with *k* = 25 per slice, selected based on sample size to balance granularity and stability. Link pruning was set to none to preserve the original network structure. Clustering was performed using the log-likelihood ratio (LLR) algorithm. The resulting cluster structure was evaluated using 2 conventional descriptive network-structure thresholds: Modularity *Q* (with *Q* > 0.3 indicating a meaningful cluster structure) and Mean Silhouette *S* (with *S* > 0.7 indicating strong within-cluster homogeneity). Q and S are descriptive indices of network structure, not inferential statistical tests; both are reported for transparency.

### 2.4. Reproducibility statement

Search was conducted on January 30, 2025 using Web of Science Core Collection (WoSCC), Science Citation Index Expanded (SCIE), and Science Citation Index (SCI) editions. Export was performed using the “Full Record and Cited References” content and “Tab-delimited (Win)” format, producing 7 export files named “savedrecs_[number].txt.” The complete search strategy was: TS=(“adenomyo*”) OR TS=(“endometriosis of the uterus”) OR TS=(“uter* endometriosis”) OR TS=(“internal endometriosis”), time span: 1980-01-01 to 2024-12-31. Analysis was performed using: Bibliometrix v4.3.0 (R v4.3.0), VOSviewer v1.6.20, and CiteSpace v6.4.R1 (Java 17).

Derived bibliometric indicators (country, institution, journal, author, and keyword indicators for each of the 3 corpora; CiteSpace network parameters per time slice and VOSviewer network thresholds) and the complete annotated R analysis script are deposited in the Open Science Framework (OSF) under DOI [10.17605/OSF.IO/GTPBQ] under a CC-BY-4.0 license. Raw WoSCC exports cannot be redistributed under the WoSCC license; the deposited package therefore contains only the derived CSV tables, the R analysis script, and the CiteSpace/ VOSviewer parameter files.

### 2.5. Patient/public involvement and ethics approval

No patients were involved in this study. The study was exempted from institutional ethics review because only publicly available data were analyzed.

## 3. Results

### 3.1. Overview of publication volume and trends

After screening, 3100 English-language articles or reviews from 609 journals and 10,689 authors, citing 53,698 references, were included in this study. The publication growth rate was 11.97% annually, with an average of 24.27 citations per paper. To assess the robustness of our findings, we performed a sensitivity analysis comparing 3 corpora: the main broad corpus (TS = search, n = 3100); a title-level restrictive corpus (adenomyosis terms in title only, n = 1645); and an adenomyosis-specific metadata corpus (adenomyosis terms in title, abstract, or author keywords, n = 3008). The 3 corpora produced substantially overlapping sets of top countries, institutions, journals, and keyword clusters, but the single top-ranked entity for several indicators differed between corpora: in the broad corpus, the leading country was China, the leading institution was Fudan University, and the leading author-name string was Sunwei Guo; in the 2 restricted corpora, the leading country was the United States, the leading institution was the University of London, and the leading author-name string was Donnez J. These differences reflect retrieval-dependent rank changes rather than contradictory findings and support the reliability of the thematic and landscape conclusions of the study (Table S3, Supplemental Digital Content 2). From 1980 to 2024, adenomyosis publications increased overall. Between 1980 and 2005, growth was slow with 475 publications (18.3 annually). After 2006, publication activity surged exponentially, resulting in 2625 publications, accounting for 84.68% of the total. Notably, the actual publication volume for the years 2021 to 2023 markedly exceeded the values predicted by the fitted curve. Average citations per paper generally rose, peaking at 3.43 in 1997, but declined steadily after 2018 (Fig. 2).

### 3.2. National publication volume and international cooperation

In the field of adenomyosis research, the top 5 countries by publication volume in the broad corpus were China (752 publications, 24.26%, full-counted), the United States (414 publications, 13.35%, full-counted), Japan (350 publications, 11.29%, full-counted), Italy (215 publications, 6.94%, full-counted), and South Korea (128 publications, 4.13%, full-counted). Notably, although China produced more publications than the US, US-based publications demonstrated higher total citations (13,002) and a higher average citation count per paper (31.40) compared to China (11,111 total citations; 14.80 average citations per paper). The top 10 countries ranked by total citation count are presented in Figure 3A. Publications were classified as either single-country publication (SCP) or multiple-country publication (MCP) based on the country of the corresponding author. Analysis revealed that the number of multinational collaborative publications was substantially lower than that of single-country publications across all nations (Fig. 3B). Although the top 10 publishing countries contributed 2306 publications, accounting for 74.39% of the total output, their average proportion of international co-authorship was only 16.14%. Regarding international collaboration, 49 countries with ≥ 5 collaborative publications formed 7 distinct clusters. The United States exhibited the strongest collaboration network (273), followed by Italy (239), the United Kingdom (189), Belgium (166), and France (124; Fig. 3C).

### 3.3. Contribution of research institutions

A total of 3102 institutions published research papers on adenomyosis, among which 102 institutions (3.29%) published 10 or more papers. The top 5 institutions by publication volume in the broad corpus were Fudan University (89 publications, 2.87%), Shandong University (52 publications, 1.68%), The University of Tokyo (49 publications, 1.58%), Capital Medical University (47 publications, 1.52%), and Chongqing Medical University (43 publications, 1.39%). Six of the top 10 publishing institutions were based in China (Table 1). It is important to note that Table 1 presents the 10 most productive institutions ranked by publication count, together with their corresponding field-specific H-indices; this is not a ranking by H-index. The field-specific H-index, a key indicator of scholarly impact within the adenomyosis research domain, was computed for each institution. In descending order, the field-specific H-indices of the 10 most productive institutions listed in Table 1 were as follows: University of Siena (28), Fudan University (27), The University of Tokyo (26), University of Roma Tor Vergata (23), Catholic University of Louvain (22), Shandong University (22), Chongqing Medical University (20), Capital Medical University (18), Shanghai Jiao Tong University (18), and Zhejiang University (17). Of these top 10, 6 are Chinese (Fudan, Shandong, Capital Medical, Chongqing Medical, Shanghai Jiao Tong, Zhejiang), 2 are Italian (Siena, Roma Tor Vergata), 1 is Japanese (Tokyo), and 1 is Belgian (Catholic University of Louvain). No German-affiliated institution is included in Table 1. Among the ten most productive institutions listed in Table 1, the 3 with the highest field-specific H-indices (Siena, Fudan, Tokyo) are therefore geographically diverse. The 3 institutions with the highest average annual citations were the University of Siena, Fudan University, and Catholic University of Louvain. Total Link Strength (TLS) values in Table 1, calculated by VOSviewer v1.6.20, are highest for Fudan University (3101), followed by University of Siena (2671), Catholic University of Louvain (1274), and Shanghai Jiao Tong University (1214).

### 3.4. Journal analysis

Out of 609 journals, 68 (11.17%) published 10 or more articles, totaling 1920 articles (61.94%). The top ten journals accounted for 732 articles (23.61%). In the field of adenomyosis research, *Fertility and Sterility*, *Human Reproduction*, and *Journal of Minimally Invasive Gynecology* ranked highest in H-index, g-index, and number of published articles. The most cited journals were *Fertility and Sterility*, *Human Reproduction*, and *American Journal of Obstetrics and Gynecology*. The top 3 impact factors for 2023 were *Human Reproduction Update*, *American Journal of Obstetrics and Gynecology*, and *Fertility and Sterility*. Regarding journal categorization, among the top ten journals by H-index, all except *Reproductive Sciences* and *European Journal of Obstetrics & Gynecology* and *Reproductive Biology* (categorized as Q2) and *International Journal of Gynecological Pathology* (categorized as Q3) fell into Q1 (Table S1, Supplemental Digital Content 3).

### 3.5. Author contributions and co-citation analysis

The top ten author-name strings with the most publications published a total of 337 articles, accounting for 10.87% of the total. There were 122 authors with 10 or more articles, with a total of 1885 articles, accounting for 60.81%. According to the H-index, the top 3 most influential authors are Sunwei Guo of Fudan University, Donnez Jacques of Catholic University Louvain, and Xishi Liu of Fudan University. Sunwei Guo, Mori Taisuke of the University of Tokyo, and Donnez Jacques are the 3 scholars with the most publications. In addition, in this field, the 3 most cited scholars are Sunwei Guo, Petraglia Felice of the University of Florence, Italy, and Exacoustos Caterina of the University of Rome Tor Vergata, Italy (Table S2, Supplemental Digital Content 4). Vercellini Paolo of the University of Milan was co-cited the most (1020), followed by Leyendecker Gerhard of Goethe University Frankfurt (649) and Bazot Marc of Sorbonne University (645). There are 315 authors with 5 or more articles, forming 20 clusters visually (Fig. S1, Supplemental Digital Content 5). Seracchioli Renato is the author with the highest cooperation strength (Total link strength 98).

### 3.6. The dynamic evolution of research topics

Document co-citation clustering analysis combined with burst detection effectively reveals the evolutionary trends of research topics and identifies emerging hotspots. The results of the co-citation clustering analysis yielded a Modularity *Q* value of 0.7713 and a mean Silhouette *S* value of 0.9008. Both values exceed the conventional descriptive thresholds (*Q* > 0.3 indicating meaningful cluster structure; *S* > 0.7 indicating strong within-cluster homogeneity); *Q* and *S* are descriptive indices of network structure, not inferential statistical tests, and are reported here for transparency (Fig. 4A).

The 3100 included publications collectively cite 53,698 references. From these, CiteSpace’s co-citation network was constructed using the parameters described above (Time slicing = 1 year, g-index with *k* = 25, link pruning = none, LLR clustering), yielding a co-citation network (Fig. 4B). The timeline visualization of co-cited literature clearly reveals the dynamic evolution of research hotspots. Research prior to 2000 primarily focused on defining the nature of adenomyosis (e.g., as a benign disorder) and establishing its fundamental concepts. During the decade leading up to 2005, the main research hotspot centered on the application of transvaginal ultrasound techniques. Regarding treatment approaches, abdominal hysterectomy, uterine artery embolization, and high-intensity focused ultrasound (HIFU) represented the primary therapeutic modalities for adenomyosis, with their research prominence demonstrating dynamic shifts over time. Fertility-preserving treatments and associated reproductive outcomes garnered significant research interest. In the domain of pathogenesis, research themes evolved from endothelial nitric oxide synthase (eNOS) and induced adenomyosis towards mechanisms involving ectopic endometrium implantation. Myometrial invasion and stromal cells emerged as key research hotspots within this context. Notably, studies investigating pregnancy outcomes began to rise in prominence around 2013, exhibited a citation burst in 2022, and continue to maintain substantial research activity. Furthermore, “consensus” (2020–2024), “pathogenesis” (2022–2024), and “pregnancy outcome” (2022–2024) are recent and currently active burst keywords, indicating their emergence as a current research focus rather than as long-standing hotspots (Fig. 5A).

### 3.7. Keyword research hotspot analysis

Keyword cluster analysis serves as an effective method for identifying research hotspots. Visualization of adenomyosis-related keywords revealed 8 distinct thematic clusters (Fig. 5B). For readability, the 4 largest clusters (#0–#3) are discussed in detail below. The largest cluster (#0) focused on the role of epithelial-mesenchymal transition (EMT) in the development of adenomyosis. Its high-frequency keywords included: “expression,” “stromal cells,” “proliferation,” “activation,” and “apoptosis.” The second-largest cluster (#1) centered on HIFU therapy for adenomyosis. Key high-frequency keywords were: “management,” “leiomyoma,” “surgery,” “artery embolization,” and “pregnancy.” The presence of “leiomyoma” in Cluster #1 reflects the clinical overlap between adenomyosis and leiomyoma in comparative studies and uterine artery embolization [UAE]/HIFU literature, rather than off-topic retrieval (see Table S4, Supplemental Digital Content 1). The third-largest cluster (#2) explored the relationship between adenomyosis and endometrial cancer. Its prominent keywords comprised: “therapy,” “estrogen,” “adenocarcinoma,” “atypical polypoid adenomyoma,” and “endometrial hyperplasia.” Cluster #3 concentrated on pregnancy outcomes in adenomyosis, characterized by high-frequency keywords: “endometriosis,” “in vitro fertilization,” “junction zone,” “risk factors,” and “infertility.” These clusters and their associated high-frequency keywords reflect the primary research hotspots and directions within the adenomyosis field, providing valuable insights for future investigations.

## 4. Discussion

### 4.1. Current status of international adenomyosis research: a bibliometric perspective

Since Rokitansky’s 1860 description of endometrial glands in the myometrium, adenomyosis research has evolved through distinct stages. From 1980 to 2005, research developed relatively slowly, concentrating on refining tools and techniques. After 2006, the field entered a phase of rapid progress due to advancements in MRI, ultrasound, and medical research techniques.^[20]^ This phase, marked by the use of innovative technologies, has greatly enhanced the understanding of adenomyosis. The present study analyzed 3100 publications from the modern bibliometric era using bibliometric analysis to highlight trends, current status, and key research areas, aiming to offer a comprehensive overview and guide future research directions.

From 2021 to 2023, publications on adenomyosis surpassed expectations, reflecting growing international interest. This rise is likely due to advancements in research tools and trends like delayed childbearing and declining fertility rates. Adenomyosis is linked to dysmenorrhea, heavy bleeding, infertility, and adverse pregnancy outcomes, highlighting its clinical and research importance. Despite increased output, citation counts per article have declined over the past 5 years. This pattern is consistent with a well-known bibliometric time-lag effect, in which recent publications have had less time to accumulate citations than older ones, rather than reflecting a decline in research quality per se. In the broad corpus, China led in publication volume; however, the US excels in total citations, international collaborations, and network strength, underscoring its leadership in the field. The sensitivity analysis further showed that the United States led both the title-level restrictive and adenomyosis-specific metadata corpora, consistent with the USA’s leading role in citation impact and international collaboration (Table S3, Supplemental Digital Content 2). International collaboration in adenomyosis research shows clear geographical trends. Besides the US, Italy, the UK, Belgium, and France, all in Europe, have strong collaborative ties. China collaborates substantially with the US and Japan but needs to enhance international engagement to reduce regional research disparities. It should be noted that country rankings use full counting, which inflates contributions from multi-country collaborations. These figures may differ from corresponding-author-based or fractional-count analyses. To advance adenomyosis research, it is crucial to strategically utilize regional strengths for high-quality, international, multicenter collaborations.

Table 1 lists the 10 most productive institutions in the broad corpus ranked by publication count, together with their corresponding field-specific H-indices (which differ from each institution’s overall H-index across all disciplines). Among the ten most productive institutions listed in Table 1, the field-specific H-indices in descending order were Siena (Italy, 28), Fudan University (China, 27), The University of Tokyo (Japan, 26), University of Roma Tor Vergata (Italy, 23), Catholic University of Louvain (Belgium, 22), and Shandong University (China, 22). The institutions with the highest field-specific H-indices are therefore geographically diverse and not dominated by a single country (Table 1). This reflects both China’s focus on scientific research and increased investment, and the substantial European and Japanese contributions to scholarly impact in the field. In journals, *Fertility and Sterility*, *Human Reproduction*, and the *Journal of Minimally Invasive Gynecology* lead in impact and volume, with *Human Reproduction Update* (UK) having the highest impact in 2023. Analyzing scholarly influence, Jacques Donnez (listed among the top 3 scholars by both H-index and g-index) has focused his research primarily on the pathophysiological mechanisms, treatment, and fertility outcomes of adenomyosis. Sunwei Guo and Xishi Liu have made significant contributions to understanding the pathogenesis, diagnosis, and treatment of adenomyosis. The research outputs of these leading scholars have not only propelled academic progress in the field but also provided crucial guidance for clinical practice.

### 4.2. Research trends and hotspots: evidence from bibliometric analysis

Adenomyosis research presents a dynamic cycle between breakthrough discoveries and new theoretical hypotheses, and the conclusion of 1 research topic often serves as a foundation for subsequent investigations rather than representing a definitive endpoint.

Therapeutic studies: For patients who have finished childbearing and for whom medical treatments have failed, hysterectomy remains the definitive option.^[21]^ Uterine artery embolization (UAE) is a safe, effective nonsurgical alternative,^[22,23]^ but only about 55% of patients maintain its benefits 2 years post-procedure, making it more suitable for short-term relief. Recurrence may necessitate further interventions, including hysterectomy.^[24]^ HIFU has emerged as a current research focus due to its noninvasive nature, showing improvement rates for menorrhagia and dysmenorrhea between 12.4% to 80.8% and 25% to 100% respectively, at 1 to 2 years posttreatment.^[25]^ However, 26% of patients experience symptom recurrence within a median of 12 months. Better outcomes are linked to a larger non-perfused volume ratio, older age, lower BMI, and higher acoustic power.^[26]^ High-quality comparative studies on HIFU’s effects on fertility and pregnancy are still needed.^[27]^ Fertility-preserving treatment for diffuse adenomyosis is challenging. The “triple-flap” technique by Osada et al.^[28]^ offers a new surgical option. The PUSH technique, which enhances suturing methods, improves fertility-sparing surgery for diffuse adenomyosis and shows promising results.^[29]^ The PUSH-technique study is authored by members of the present author group and is the only published outcome study of this fertility-preserving procedure for diffuse adenomyosis. However, long-term efficacy and impact on pregnancy outcomes require further validation.

Pathogenesis studies: Early research primarily focused on eNOS^[30]^ with an average study duration in 1996. Dysregulated eNOS expression can impact endometrial receptivity and menstrual bleeding regulation.^[31]^ Animal models are crucial for studying pathogenesis and treatment. For instance, mouse models have shown that prolactin and ovarian steroid hormones contribute to adenomyosis when induced by intrauterine pituitary allotransplantation.^[32]^ Antiplatelet therapy has been effective in reducing myometrial invasion and symptoms in models where tamoxifen is administered to neonatal mice.^[33]^ Additionally, adenomyosis can be induced by mechanical or thermal damage to the endometrial-myometrial interface, which can be mitigated by using neurokinin 1 receptor (NK1R) antagonists or β-blockers perioperatively.^[34]^ Molecular studies show significant differences in gene expression between ectopic and eutopic endometrium, involving various mRNAs and lncRNAs.^[35]^ Abnormal metabolism and cytokine expression in these tissues can promote angiogenesis, cell proliferation, prevent apoptosis, enhance local estrogen effects, block progesterone actions, and increase endometrial infiltration and ectopic growth.^[36]^ EMT has become a popular research topic recently. EMT is a conserved process that converts immobile epithelial cells into mobile stromal cells^[37]^ and is crucial in adenomyosis development.^[38]^ Estrogen-induced EMT enhances cell migration and invasion, allowing endometrial epithelial cells to penetrate the myometrium.^[39]^ Additionally, increased platelet aggregation within adenomyotic lesions contributes to fibrosis by promoting EMT, fibroblast-to-myofibroblast transdifferentiation (FMT), and smooth muscle metaplasia, thereby participating in lesion formation.^[40]^ Macrophage and endometrial cell interactions can also induce EMT-like processes.^[41]^ Although research continues, the exact cause of adenomyosis is not fully understood. Two main theories exist: The Endometrial Invagination Theory suggests that high estrogen levels or increased estrogen receptor activity at the endometrial-myometrial interface (EMI), possibly in conjunction with the oxytocin/oxytocin receptor system, lead to tissue damage and repeated repair. This process damages the EMI, enabling endometrial tissue to invade and develop into adenomyosis.^[38]^ Adenomyosis develops from the metaplasia of Müllerian duct remnants or the migration and differentiation of endometrial stem cells into the myometrium.^[38,42]^ Local hyperestrogenism and progesterone resistance play critical roles in its development and progression.^[43]^ The pathogenesis involves a complex interplay of mechanisms including sex hormone receptor signaling, inflammatory responses, neuroangiogenesis, fibrosis,^[42]^ EMT,^[38]^ and genetic and epigenetic modifications.^[44]^ Since 2022, “pathogenesis” has emerged as a burst keyword due to advancements in detection technology and evolving understanding,^[45]^ highlighting increased focus and anticipated breakthroughs in disease mechanism research.

Adenomyosis and pregnancy outcomes: Recent attention has focused on adenomyosis’s negative impact on in vitro fertilization (IVF) outcomes and its link to obstetric complications. Substantial evidence shows adenomyosis lowers IVF pregnancy and live birth rates and raises miscarriage risks^[46,47]^ due to defects in the endometrium and myometrium, impaired uterine peristalsis, reduced endometrial receptivity, and high free radical levels within the uterine environment.^[48]^ It is also associated with various adverse pregnancy outcomes like second-trimester pregnancy loss, placenta malposition,^[49]^ preterm birth, premature rupture of fetal membranes, small for gestational age infants,^[50]^ hypertensive disorders, cesarean delivery, and postpartum hemorrhage,^[51]^ with diffuse adenomyosis posing a higher risk than the focal form.^[51]^ Global fertility rates have been declining, with the total fertility rate dropping from 3.3 in 1920 to 2.3 in 2021, nearing the replacement level of 2.1. This trend calls for policies to support family formation and childbirth.^[52]^ While the link between adenomyosis and negative pregnancy outcomes is well-documented, the mechanisms are not fully understood.^[52,53]^ Analysis of keyword clustering and burst keyword detection indicates that the relationship between adenomyosis and pregnancy outcomes is not only a current research hotspot but also a critical future research direction.

Association with endometrial cancer: Adenomyosis can occur alongside endometrial carcinoma, with a 28% co-occurrence rate in hysterectomy specimens and malignant transformation observed in about 11% of cases.^[54]^ Endometrial cancer arising in adenomyosis (EC-AIA) differs from cancer simply coexisting with adenomyosis (EC-A) by affecting older patients, showing more non-endometrioid subtypes, having deeper myometrial invasion, and leading to a poorer prognosis. EC-AIA is often confined to the myometrium (63.6%) without involving the contiguous endometrium.^[55]^ Although some studies suggest that concurrent adenomyosis might be a favorable prognostic factor in endometrial cancer,^[56,57]^ the literature on this association remains controversial.^[58]^ Adenomyosis and endometrial carcinoma share commonalities in both pathogenesis and treatment. Current treatments, including progesterone therapy and surgery, require long-term management.^[59]^ However, the molecular mechanisms underlying malignant transformation within adenomyotic foci are still poorly understood. Further research is essential to elucidate the factors driving disease progression and influencing prognosis^[54,60]^ and to develop more effective long-term therapeutic strategies based on these insights.

Diagnostic classification challenges: Due to the high heterogeneity of adenomyosis and its frequent co-occurrence with other conditions, there is currently a lack of internationally standardized terminology, diagnostic criteria, and classification systems.^[9,61]^ To address this gap, a conference abstract/presentation addressing classification and diagnosis, summarizing current classification and diagnostic approaches for adenomyosis in women of reproductive age, was presented at the 2024 ESHRE Annual Meeting by representatives of the European Society for Gynaecological Endoscopy (ESGE), the European Society of Human Reproduction and Embryology (ESHRE), and the World Endometriosis Society (WES).^[62]^ This conference abstract notes that although several classification systems based on Morphological Uterus Sonographic Assessment (MUSA)^[63]^ and Magnetic Resonance Imaging (MRI)^[13]^ have been proposed, none have yet achieved international consensus or undergone rigorous clinical validation, and inconsistencies between these different approaches add complexity to the diagnostic process. The conference abstract focuses on establishing standardized terminology, classification, and diagnostic pathways for adenomyosis in reproductive-aged women, reviews existing classification systems based on histology, ultrasound, and MRI findings, and provides diagnostic recommendations, thereby laying a foundation for subsequent management and surgical planning. It explicitly acknowledges that due to the current lack of robust evidence, its recommendations primarily rely on clinical expertise. The establishment of internationally unified diagnostic criteria, evidence-based classification systems, and optimized treatment regimens – particularly those focused on fertility preservation and improving pregnancy outcomes – therefore requires continued and focused exploration, and a formal joint guideline from these societies is still in development.

### 4.3. Robustness of findings across retrieval strategies

To evaluate whether our bibliometric conclusions are sensitive to the choice of retrieval strategy, we performed a sensitivity analysis comparing 3 corpora derived from the same base query (Table S3, Supplemental Digital Content 2). The Broad Corpus (n = 3100) reflects all adenomyosis-related records retrieved by the WoSCC query. The Title-Level Restrictive Corpus (n = 1645) further required “adenomyosis” to appear in the article title, while the Adenomyosis-Specific Metadata Corpus (n = 3008) required the term to appear in at least one of the title, abstract, or keyword fields. All 3 corpora were retrieved and analyzed concurrently to ensure internal consistency.

The 3 corpora produced substantially overlapping sets of top indicators but differed in the single top-ranked entity for several indicators. The top country was China in the Broad Corpus and the United States in both the Title-Level Restrictive and Adenomyosis-Specific Metadata corpora. The top institution was Fudan University in the Broad Corpus and the University of London in both restricted corpora. The top author-name string was Sunwei Guo in the Broad Corpus and Donnez J. in both restricted corpora. The top journal was *Fertility and Sterility* in both the Broad Corpus and the Title-Level Restrictive Corpus, but *Human Reproduction* ranked first in the Adenomyosis-Specific Metadata Corpus, with *Fertility and Sterility* ranking second in that corpus. The dominant keyword (“adenomyosis”) was consistent across all 3 corpora. The overlapping leadership sets across progressively narrower retrieval conditions support the robustness of the principal landscape findings of the present study, while the retrieval-dependent changes in the single top-ranked entity indicate that headline rankings should be interpreted in the context of the retrieval strategy used.

The Title-Level Restrictive Corpus showed a substantially higher average citation count per article (33.35) than the Broad Corpus (24.27), suggesting that articles explicitly featuring “adenomyosis” in the title tend to be more focused and influential. In contrast, the Adenomyosis-Specific Metadata Corpus yielded citation indicators (24.43 per article) virtually identical to those of the Broad Corpus, indicating that the inclusion of abstract- and keyword-level coverage captures nearly the full body of relevant work and is unlikely to materially alter the conclusions. The USA’s leading position in the 2 restricted corpora is consistent with the USA’s leading total citations (13,002) and average citation per paper (31.40) reported in the main analysis.

### 4.4. Limitation

This bibliometric analysis provides valuable insights into adenomyosis research; however, several limitations warrant consideration. First, while the WoSCC is one of the most comprehensive and authoritative databases, publications from non-indexed journals not covered by WoSCC were excluded from this study. Furthermore, non-English publications were also not included, which may introduce a degree of selection bias. Our sensitivity analysis across 3 corpora confirmed that the major thematic and landscape findings were robust across corpora, while the single top-ranked entity for several indicators differed by retrieval strategy. The citation and publication volume metrics reflect the broader indexed literature. Second, due to the extensive time span of the literature search, some earlier publications lacking keywords, abstracts, or full-text information were manually excluded during the screening process, potentially compromising comprehensiveness. Third, although manual standardization of certain terminology was performed prior to analysis to minimize errors arising from variations in expression, potential inaccuracies cannot be entirely ruled out. Fourth, the 82% hysterectomy figure cited in the Introduction is specific to a US insured-population cohort (Kaiser Permanente Washington, 2006–2015) and may not generalize to other healthcare systems or uninsured populations. Fifth, protocol registration was not performed prior to this study, which limits the transparency of the methodological design. Future bibliometric reviews should consider prospective registration on platforms such as PROSPERO or OSF to enhance methodological transparency and reduce reporting bias.

## 5. Conclusion

This scientometric study systematically analyzed adenomyosis literature from 1980 to 2024, providing comprehensive insights. Despite rapid research advancements, average citations per article have decreased over the past 5 years; this trend most likely reflects a bibliometric time-lag effect, in which recent publications have had less time to accumulate citations than older ones, rather than a decline in research quality. Furthermore, just 16.14% of publications from the top ten productive countries involved international collaboration, highlighting an opportunity for expanded international collaboration. In the broad retrieval corpus, China led in publication volume; in the 2 adenomyosis-specific retrieval corpora, the United States ranked first. Current research hotspots primarily focus on the role of ectopic endometrium and EMT in the pathogenesis, the fertility outcomes and long-term efficacy of treatments like HIFU and fertility-sparing surgeries, the association with endometrial cancer, and the link with adverse pregnancy outcomes. “Consensus,” “pathogenesis,” and “pregnancy outcome” are recent and currently active burst keywords (2020–2024 and 2022–2024), signaling the field’s emerging priorities. In addition to deepening research on pathogenesis and pregnancy outcomes, key future directions include enhancing international collaboration and establishing internationally agreed, evidence-based consensus on standardized terminology, diagnostic criteria, and classification systems for adenomyosis. Future studies should also link bibliometric trends to clinical trial registries, guideline development timelines, and real-world treatment data to determine whether publication trends correspond to measurable changes in clinical research priorities or patient care.

## Author contributions

**Conceptualization:** Ruifang Wu, Xiuli Sun.

**Data curation:** Qicai Hu, Liping Zeng.

**Formal analysis:** Qicai Hu, Liping Zeng, Chunlei Guo.

**Funding acquisition:** Ruifang Wu, Xiuli Sun.

**Investigation:** Qicai Hu, Liping Zeng, Chunlei Guo, Chunying Li.

**Methodology:** Qicai Hu, Chunlei Guo, Chunying Li.

**Writing – original draft:** Qicai Hu, Liping Zeng, Chunlei Guo.

**Writing – review & editing:** Ruifang Wu, Xiuli Sun.










