# Supplementary material for: Bibliometric analysis and visualization of adenomyosis: From 1980–2024
Source: Medicine (Baltimore). 2026 Oct 16;105(42):e51082. doi: 10.1097/MD.0000000000051082 (PMC13680950; doi:10.1097/MD.0000000000051082)
Supplement: Supplementary file 1 [file medi-105-e51082-s001.pdf]

Supplemental material

Prior to keyword co-occurrence analysis, a harmonization step was performed using VOSviewer's thesaurus function.

Table S4 Excluded and Adjacent-Topic Terms in Keyword Analysis (excluded background terms and retained adjacent-topic terms with rationale)

Category	Term(s)	Reason
Excluded (background noise)	women, patient, human, female, uterus, uterine	Generic demographic/anatomical terms that appear universally
Adjacent-topic (retained, flagged)	leiomyoma, endometriosis	Related but distinct disease entities; leiomyoma frequently appears in adenomyosis comparative studies and UAE/HIFU literature, reflecting genuine clinical overlap rather than off-topic retrieval. Interpretations in cluster context are provided in Results Section 3.7.

Note: 'Leiomyosarcoma' was not present in our adenomyosis keyword corpus and therefore does not appear in this table.
