# Supplementary material for: Bibliometric analysis and visualization of adenomyosis: From 1980–2024
Source: Medicine (Baltimore). 2026 Oct 16;105(42):e51082. doi: 10.1097/MD.0000000000051082 (PMC13680950; doi:10.1097/MD.0000000000051082)
Supplement: Supplementary file 2 [file medi-105-e51082-s002.pdf]

Supplemental material

To assess the robustness of our findings, we performed a sensitivity analysis comparing three corpora: (1) the main broad corpus (TS= search, n=3,100); (2) a title-level restrictive corpus (adenomyosis terms in title only); and (3) an adenomyosis-specific metadata corpus (adenomyosis terms in title, abstract, or author keywords).

Note: The Title-Level Restrictive Corpus includes only records where adenomyosis-related terms appear in the article title. The Adenomyosis-Specific Metadata Corpus includes records where such terms appear in title, abstract, or author keywords.

Table S3 Sensitivity Analysis: Comparison of Three Corpora (Broad, Title-Level Restrictive, Adenomyosis-Specific Metadata) - country, institution, journal, author, keyword indicators, H-index, total citations, and average citation per paper

Metric	Broad Corpus (n=3,100)	Title-Level Restrictive Corpus	Adenomyosis-Specific Metadata Corpus
Records	3,100	1,645	3,008
Total citations	75,237	54,861	73,485
Average citations/article	24.27	33.35	24.43
Top country	China (752, 24.26%)	USA (372, 22.6%)	USA (701, 23.3%)
Top institution	Fudan University (89)	University of London (25)	University of London (28)
Top journal	Fertility and Sterility	Fertility and Sterility	Human Reproduction
Top keyword	adenomyosis / EMT cluster	adenomyosis	adenomyosis

All three corpora were retrieved and analyzed concurrently from the Web of Science Core Collection; minor variations in indicators across corpora reflect differences in retrieval scope rather than temporal drift in citation accumulation.
