# Supplementary material for: Bibliometric analysis and visualization of adenomyosis: From 1980–2024
Source: Medicine (Baltimore). 2026 Oct 16;105(42):e51082. doi: 10.1097/MD.0000000000051082 (PMC13680950; doi:10.1097/MD.0000000000051082)
Supplement: Supplementary file 3 [file medi-105-e51082-s003.pdf]

Supplemental material

**Table S1** H-index top 10 journals, records, percentage, g-index, LCS, IF-2023, JCR(2023) and total number of citations.

| Rank | Source | Records | Initial year | Percentage(n/3100,%) | H-index | g-index | LCS | IF 2023 | JCR(2023) | Total number of citations |
|---|---|---|---|---|---|---|---|---|---|---|
| 1 | FERTILITY AND STERILITY | 139 | 1993 | 4.48 | 57 | 90 | 10174 | 6.6 | Q1 | 8783 |
| 2 | HUMAN REPRODUCTION | 82 | 1987 | 2.65 | 43 | 79 | 6367 | 6 | Q1 | 6258 |
| 3 | JOURNAL OF MINIMALLY INVASIVE GYNECOLOGY | 88 | 2005 | 2.84 | 31 | 48 | 2577 | 3.5 | Q1 | 2662 |
| 4 | HUMAN REPRODUCTION UPDATE | 29 | 1998 | 0.94 | 26 | 29 | 2599 | 14.8 | Q1 | 4144 |
| 5 | BEST PRACTICE & RESEARCH CLINICAL OBSTETRICS & GYNAECOLOGY | 34 | 2000 | 1.10 | 24 | 34 | 1774 | 3.9 | Q1 | 2211 |
| 6 | AMERICAN JOURNAL OF OBSTETRICS AND GYNECOLOGY | 34 | 1980 | 1.10 | 23 | 34 | 4398 | 8.7 | Q1 | 1736 |
| 7 | REPRODUCTIVE SCIENCES | 65 | 2008 | 2.10 | 23 | 36 | 1491 | 2.6 | Q2 | 1505 |
| 8 | INTERNATIONAL JOURNAL OF GYNECOLOGICAL PATHOLOGY | 40 | 1985 | 1.29 | 21 | 36 | 1063 | 1.6 | Q3 | 1357 |
| 9 | REPRODUCTIVE BIOMEDICINE ONLINE | 55 | 2007 | 1.77 | 21 | 41 | 1769 | 3.7 | Q1 | 1730 |
| 10 | EUROPEAN JOURNAL OF OBSTETRICS & GYNECOLOGY AND REPRODUCTIVE BIOLOGY | 60 | 1980 | 1.94 | 20 | 36 | 1744 | 2.1 | Q2 | 1463 |

LCS, local citation scores; IF, impact factor; JCR, Journal citation reports.
