# Supplementary material for: Bibliometric analysis and visualization of adenomyosis: From 1980–2024
Source: Medicine (Baltimore). 2026 Oct 16;105(42):e51082. doi: 10.1097/MD.0000000000051082 (PMC13680950; doi:10.1097/MD.0000000000051082)
Supplement: Supplementary file 4 [file medi-105-e51082-s004.pdf]

Supplemental material

Table S2 Top 10 most active authors ranked by highest H-index

Rank	Author	Articles	H-index	g-index	Total-citations	LCS	Average citation per year	Institutions	Articles fractionalized
1	Guo Sunwei	46	25	42	1842	1223	115	Fudan University	16.18
2	Donnez Jacques	37	24	37	3358	617	116	Catholic university louvain	10.36
3	Liu Xishi	36	20	33	1135	750	71	Fudan University	10.85
4	Brosens Ivo	21	19	21	1634	781	50	Katholieke University Leuven	6.66
5	Chapron Charles	30	18	30	1415	861	64	Universite Paris Cite	3.71
6	Exacoustos Caterina	33	18	33	1833	1038	141	University of Rome Tor Vergata	4.3
7	Benagiano Giuseppe	26	17	26	1393	773	42	Catholic University of the Sacred Heart	8.4
8	Mori Taisuke	45	17	31	1035	564	24	University of Tokyo	11.05
9	Petraglia Felice	29	17	29	1875	1146	117	University Florence	4.64
10	Zupi Errico	29	17	29	1279	727	98	University of Bologna	3.86

LCS, local citation scores.
