# Supplementary material for: Bibliometric analysis and visualization of adenomyosis: From 1980–2024
Source: Medicine (Baltimore). 2026 Oct 16;105(42):e51082. doi: 10.1097/MD.0000000000051082 (PMC13680950; doi:10.1097/MD.0000000000051082)
Supplement: Supplementary file 5 [file medi-105-e51082-s005.pdf]

Supplemental material

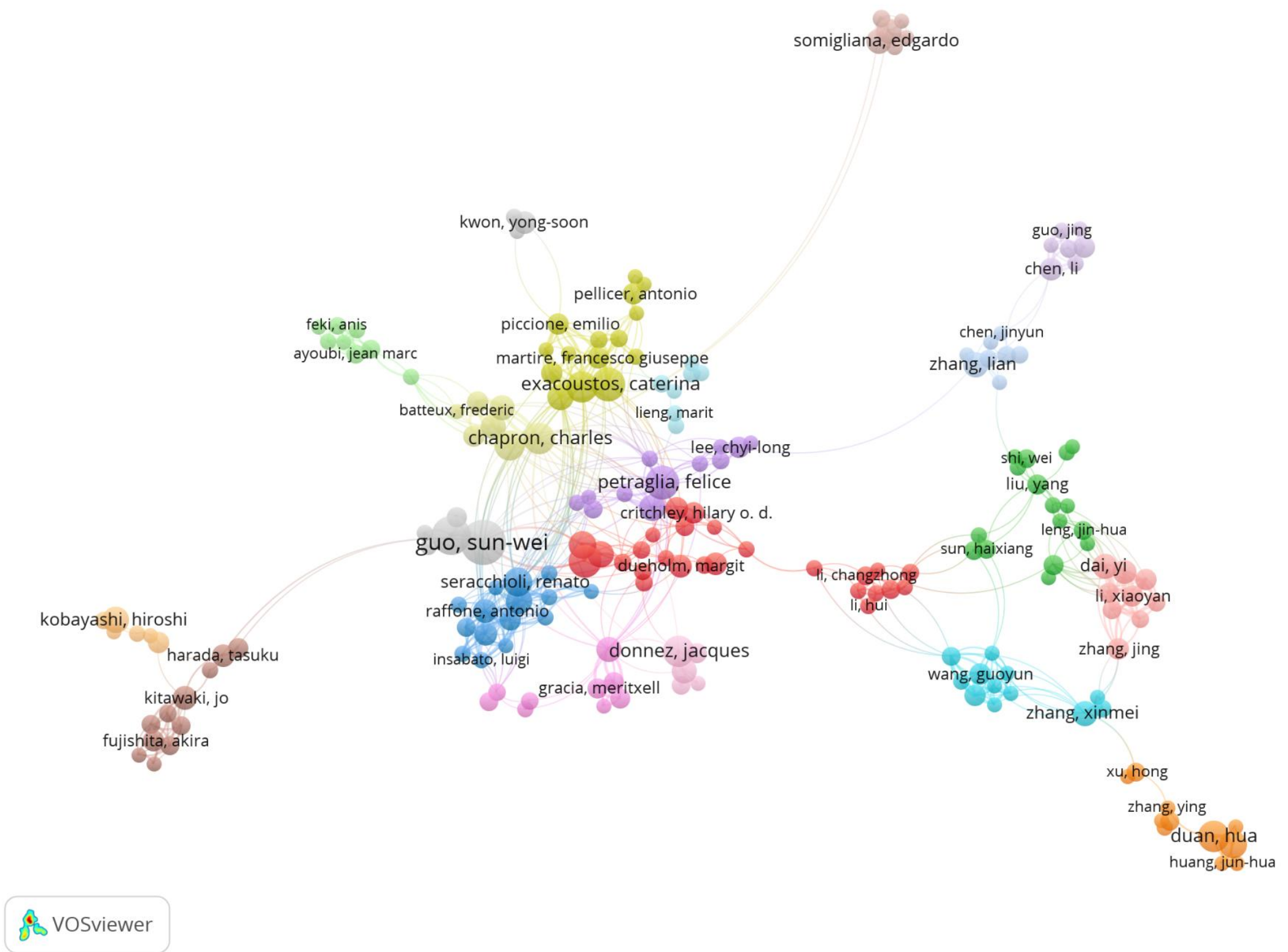

Supplemental Figure S1 Visual analysis of authors with five or more cooperative publications.
